# Unprotected anal Intercourse among Iranian Intra-Venous Drug Users

**DOI:** 10.3389/fpubh.2013.00034

**Published:** 2013-09-26

**Authors:** Parvaneh Mirabi, Mosaieb Yarmohmmadi Vasel, Babak Moazen, Mahmoud Sehat, Majid Rezazadeh, Khodabakhsh Ahmadi

**Affiliations:** ^1^Department of Midwifery, Zanjan Branch, Islamic Azad University, Zanjan, Iran; ^2^Department of Psychology, Bu-Ali Sina University, Hamedan, Iran; ^3^Medicine and Health Promotion Institute, Tehran, Iran; ^4^Universal Network for Health Information Dissemination and Exchange (UNHIDE), Tehran, Iran; ^5^AIDS Prevention and Control Committee, Welfare Organization State, Tehran, Iran; ^6^Behavioral Sciences Research Center, Baqiyatallah Medical Sciences University, Tehran, Iran

**Keywords:** risk behaviors, unprotected sex, anal intercourse, Iran, intra-venous drug users

## Abstract

**Purpose:** To assess the prevalence and associated factors of unprotected anal intercourse among Iranian male heterosexual Intra-Venous Drug Users (IDUs).

**Methods:** In a cross-sectional study 360 male heterosexual IDUs were sampled from streets of eight different geographical parts of Iran. Variables such as socio-demographics, HIV knowledge (10 items), and HIV attitude (16 items) were entered to a logistic regression to determine the predictors of unprotected anal intercourse during the past month.

**Results:** From all, 20.8% reported unprotected anal intercourse during the past month. HIV knowledge was not significantly different among IDUs with and without unprotected anal intercourse. High age [odds ratio (OR) = 0.954, 95% confidence intervals (CI) = 0.916–0.992] was associated with a lower likelihood of unprotected anal intercourse, while being not married (OR = 2.301, 95% CI = 1.151–4.601), and high perceived HIV risk (OR = 1.776, 95% CI = 1.376–2.290) were associated with a higher likelihood of unprotected anal intercourse.

**Conclusion:** Although the results might not be generalizable to all Iranian IDUs, this study findings may still be helpful for design and implementation of public health programs in Iran to prevent sexual transmission of HIV through IDUs.

## Introduction

People who engage in unprotected sex may have different motivations. Some people may want to show intimacy, romance, and trust to a sex partner through practicing unprotected sex (1). Some other people may decline to use condom to express their masculinity and individual choice. Other individuals may practice unprotected sex to achieve more sexual pleasure (2). Making more money is another possible reason for practice of unprotected sex among sex workers (3).

Considerable evidence has suggested that likelihood of HIV transmission by unprotected anal intercourse is several times higher than through unprotected vaginal intercourse (4). Although anal intercourse might be less common than oral or vaginal sex (6), it is still a common sexual mode among heterosexual couples (5).

Having more sex partners (10) and shared injecting (11) introduce Intra-Venous Drug Users (IDUs) as a group high risk for HIV and AIDS (7–9). Through sex with general population, injecting drug users become a source for HIV transmission to the community. This may spread HIV from a concentrated epidemic to a non-concentrated one. Unfortunately, current harm reduction policies have failed to decrease sexually transmitted infections prevalence among IDUs (11).

Size of HIV infections and IDUs are increasing rapidly in Iran (12). Unfortunately, there are very few studies assessing high-risk sexual behaviors among Iranian IDUs. Much less has been done to determine the prevalence and associated factors of unprotected anal intercourse among male IDUs in Iran.

The current study aimed to assess prevalence and associated factors of unprotected anal intercourse among Iranian male heterosexual injecting drug users.

## Materials and Methods

### Design and setting

With a cross-sectional design, this study was a secondary analysis of a data set of IDUs in eight different geographical parts of Iran: Tehran, Shiraz, Esfahan, Arak, Ahvaz, Rasht, Mashhad, and Ardebil. The survey with the unique sample was conducted by the Behavioral Sciences Research Center, Baqiyatallah University of Medical Sciences during 2009. Other reports have been extracted from this data set focusing on causes of condom non-use and syringe sharing (11, 12, Rezazade and Ahmadi, under review).

### Ethical procedures

Informed consent was obtained from all participants after they were verbally assured that the information would be kept confidential, especially from the correctional setting. All checklists and questionnaires were anonymous. Study protocol was approved by the ethical review committee of the Baqiyatallah University (13, Ahmadi and Rezazade, accepted).

### Participants and sampling

Participants were male IDUs recruited from streets in the above listed cities by snowball sampling over a 7 month period in 2009. Every participant who reported at least one drug injection during his life time was eligible. Heterosexuality was cleared by this question: “How many of your sexual partners were male and how many were female”? Those who reported sex with female(s) during their life time and declined sex with male partner(s) were considered as heterosexual.

### Process

Structured interviews were conducted by research assistants who were trained through workshops. Each interview lasted up to 60 min. No financial incentives were offered to the participants, however, condom and sterile injection equipment were delivered as compensation.

### Measures

Our questionnaire included socio-demographic data (i.e., age, gender, marital status, educational level, housing, and occupational situation), family data (i.e., number of siblings, number of children, living status, and parental living status), history of childhood trauma (i.e., experiencing violence by family members, level of family intimacy), HIV attitude (16 items such as perceived HIV risk, eagerness to learn about HIV, changing some behaviors such as drug use and sexual habits, self efficiency about high-risk behaviors), HIV knowledge (10 items about condom use, sex with a healthy looking person, HIV transmission routs), and sexual behaviors (including number of sexual intercourses, number of unprotected sexual acts, number of sex with drug users and injectors, and reason of condom non-use during the past 1 and 6 months).

### Main outcome

The following single item was applied to determine unprotected anal intercourse: “During the past month, how many times had you unprotected anal sex”? Similar outcome has been assessed using one question, before (14, 15). We dichotomized the outcome to having and not having any unprotected anal sex.

### Statistical analysis

The data obtained in the Statistical Package for the Social Sciences 17 (SPSS Inc., IL, USA) for Windows. For bi-variate analysis, Man Whitney and Chi-square tests were used. Logistic regression model was applied to determine associated factors of unprotected anal intercourse during the past month. Odds Ratios (ORs) and 95% Confidence Intervals (CI) are reported. *p*-Value <0.05 considered significant.

## Results

Of the 360 IDUs who included in this study, 55% were single, 93.1% had at least primary education, 7.8% reported living at friends or relative’s house, 34.7% were unemployed, and 19.2% reported living alone. Thirty one point two percent reported themselves being exposed to intensive violence by their family members (much and too much), and 33.1% thought that will never acquire HIV in their life time, and 58.6% had never participated in educational HIV prevention courses (Tables 1 and 2).

**Table 1:** **Socio-demographic data among Iranian heterosexual male IDUs (*n* = 360)**.

Data	*n*	%
**SOCIO-DEMOGRAPHIC DATA**		
Marital status
Single	198	55.0
Married	158	43.9
Missing	4	1.1
Educational level
Uneducated	25	6.9
Primary school	74	20.6
Some secondary school	187	51.9
High school diploma	51	14.2
Associated degree	18	5.0
Bachelor’s degree and higher	2	0.6
Missing	3	0.8
Housing
Personal	94	26.1
Rental	73	20.3
Father’s house	139	38.6
Friend’s house	18	5.0
Relatives house	10	2.8
Missing	26	7.2
Occupational status
Student	6	1.7
Self-employe	174	48.3
Employe	4	1.1
Retired	3	0.8
Homemaker	2	0.6
Home service	10	2.8
Work at private companies	14	3.9
Unemployed	125	34.7
Missing	14	3.9
Living status
Alone	72	20.1
With family members	260	82
With others	26	7.3
Missing	2	0.6

**Table 2:** **Descriptive statistics of childhood trauma and HIV attitude among male Iranian heterosexual IDUs (*n* = 360)**.

	*n*	%
**CHILDHOOD TRAUMA**		
Exposure to violence
Never	58	16.1
Little	76	21.1
Sometimes	108	30.0
Much	56	15.6
Too much	56	15.6
Missing	6	1.7
**HIV ATTITUDE**		
Perceived HIV risk
Not at all	119	33.1
A little	97	26.9
Some	93	25.8
High	27	7.5
Very high	17	4.7
Missing	7	1.9
Participation in HIV prevention courses
Never	211	58.6
Once	65	18.1
Twice	30	8.3
More	48	13.3
Missing	6	1.7

Seventy five heterosexual IDUs (20.8%) reported at least one anal intercourse during the past month. In bi-variate analysis, being non-married (OR = 0.404, 95% CI = 0.231–0.706) was associated with an increased likelihood of this outcome. Median perceived HIV risk was significantly higher among those with unprotected anal intercourse in comparison with those without this behavior (Median = 3, Q1 = 2, Q3 = 4 vs. Median = 2, Q1 = 1, Q3 = 3, *p* < 0.001).

HIV knowledge was not significantly different among IDUs with and without unprotected anal intercourse (Median = 5, Q1 = 2, Q3 = 7 vs. Median = 5, Q1 = 2, Q3 = 7, *p* = 0.653). In logistic regression, lower age (OR = 0.954, 95% CI = 0.916–0.992), being not married (OR = 2.301, 95% CI = 1.151–4.601), and higher perceived HIV risk (OR = 1.776, 95% CI = 1.376–2.290) and were associated with higher likelihood of unprotected anal intercourse (Table 3).

**Table 3:** **Predictors of unprotected anal intercourse based on a logistic regression among Iranian male heterosexual IDUs**.

Variable	*df*	Exp (B)	95% CI for exp (B)	
			Upper	Lower
Age	1	0.954	0.916	0.992
Not married	1	2.301	1.151	4.601
Perceived HIV risk	1	1.776	1.376	2.290

## Discussion

About 21% of Iranian heterosexual IDUs report at least one unprotected anal sex during the past month; however higher age, being unmarried, and higher perceived HIV risk were associated with a higher prevalence of this behavior. This study sheds light to a less researched area of sexual behavior of IDUs, the unprotected anal sex. Existing literature has mostly focused on vaginal intercourse and its associated factors, rather than other types of sex.

This considerable amount of unprotected anal intercourse reported by IDUs might be explained by the fact that some people even don’t consider anal sex as sex, and if they think that anal sex is not sex, they may not feel need to condom use. It is notable that practice of heterosexual anal intercourse is highly affected by culture and ethnicity (16). Anal intercourse among IDUs especially heterosexual IDUs needs more research.

Being not married (OR = 2.301) increased the likelihood of unprotected anal intercourse among Iranian heterosexual IDUs. Most of studies (17, 18) but not all (19) confirms our finding due to deviant, substance-abusing sources, and having multiple sex partners as well as offering an economic security and social integration to non-married people. In general population, well designed studies have shown considerable positive effects of marriage on health behaviors (18). This possible health impact has been explained by two theories. “Selection theory” suggesting healthier people more likely to get married and “protection theory” talking about providing a shield against health risk behaviors (20). However negative effects of being married on drug outcomes have been explained with this term: “If one side of a couple has drug problems, it is likely that other side has problems too” (17).

We found more unprotected anal intercourse among younger IDUs (OR = 0.945 for age). In terms of HIV infection, most studies have suggested younger people as more vulnerable group. For all types of sex, a lower frequency is expected to occur in higher ages, and anal sex is not an exception. Sexual desire is known to decrease by aging (22). This however does not mean that older IDUs do not need attention for their sexual risk behaviors. Anti-impotence and erectile-dysfunction drugs has extended the sex life to higher age and may buffer age related sexual limitations (21). Some researchers believe that “older people become sexually active in a world where there were no AIDS” and compare to younger people, they might be less aware of the consequences of unprotected sex, making them less accustomed to using condoms (21).

Unexpectedly, higher perceived HIV risk (OR = 1.776) was associated with a higher likelihood of unprotected anal intercourse among Iranian IDUs. There are contradictory findings about association of perceived HIV risk and high-risk behaviors. Although most scholars have suggested more preventive practices by increasing perceived HIV risk (23, 24), interestingly some others have shown opposite results (25), which is in line with our finding. This controversy may be caused by measurement incompatibility, subpopulation and behavioral distinctions, and unexamined critical factors constructing perceived risk (25).

In our study, HIV knowledge did not predict unprotected anal intercourse among heterosexual Iranian IDUs. Most studies haven’t entered knowledge as a possible predictor, or have found no association in this regard (26–28). Among that few studies which have reported a relation between awareness level and high-risk behaviors (29, 30), one has not even controlled the effect of confounding variables (30). Literature also has documented no impact of merely media campaigns on HIV risk behavior (31). These evidences weaken the role of HIV knowledge promotion programs in prevention of HIV infection through decreasing high-risk behaviors among IDUs.

Results of this study and some others (21) highlight the need of more attention to older IDUs as a high-risk group which raises the risk of HIV infection and other sexual transmitted diseases among uninfected people. As previously suggested (26), our study confirms that among existing factors which possibly affect HIV transmission, HIV knowledge seems less important. We suggest policy makers to focus on socioeconomics variables such as marital status, as it has been suggested by other researchers before (26–28). All in all, our findings should be implemented as a part of “combined prevention programs” (32).

Considerable amount of missing data, including 4% no answer unprotected anal intercourse is one of the limitations of this study. Being cross-sectional, causative associations were not conclusive. Under-reporting is a common consequence of self-reported data collection due to social desirability bias or concerns of confidentiality (33). We were not able to assess relationship between injection frequency and our outcome. The study was also limited to measure individual level factors (34). Current study only enrolled male IDUs. We already know that there are important gender differences in sexual behaviors, substance use, and their correlated factors (35–40).

However, two reasons represent our findings more important, firstly the stressed role of male partner in transmission of HIV because of their more high-risk sexual behaviors in comparison with females (41), and secondly, more probability of HIV transmission due to anal intercourse than vaginal intercourse (2). Thus, the study is hoped to provide a better picture from substance use (42–47) and blood born infections (48–55) and their associated factors in developing countries such as Iran.

Despite efforts to better understand HIV risk profile of IDUs in Iran, what we do not know is much more than what we know (56–64). Further research should explore profile of risk behaviors that result in transmission of HIV and other blood born infections. Such research also provides information about how substance use and sexual behavior influence wellbeing and quality of life of IDUs (65, 66).

To conclude, about 21% of Iranian male heterosexual IDUs report unprotected anal intercourse during the previous month. Iranian male IDUs who are younger, not married, and report higher HIV perceived risk are at higher risk of engagement in unprotected anal intercourse.

## Conflict of Interest Statement

The authors declare that the research was conducted in the absence of any commercial or financial relationships that could be construed as a potential conflict of interest.
